# Radiological assessment of the dissection area in supraomohyoid neck dissection

**DOI:** 10.1007/s00276-024-03453-3

**Published:** 2024-08-09

**Authors:** Yohei Takeshita, Joe Iwanaga, Yoshio Ohyama, Soichiro Ibaragi, Yuki Matsushita, R. Shane Tubbs, Norio Kitagawa, Toshiyuki Kawazu, Miki Hisatomi, Shunsuke Okada, Mamiko Fujikura, Junichi Asaumi

**Affiliations:** 1 Department of Oral and Maxillofacial Radiology, Faculty of Medicine, Dentistry and Pharmaceutical Sciences, Okayama University, 2-5-1 Shikata-cho, Kita-ku, Okayama, 700-8558 Japan; 2 Clinical Anatomy Research Association in Oral and Maxillofacial Surgery, Fukuoka, Japan; 3 Department of Oral and Maxillofacial Anatomy, Graduate School of Medical and Dental Sciences, Tokyo Medical and Dental University, Tokyo, Japan; 4 Department of Neurosurgery, Tulane Center for Clinical Neurosciences, Tulane University School of Medicine, New Orleans, LA USA; 5 Department of Neurology, Tulane Center for Clinical Neurosciences, Tulane University School of Medicine, New Orleans, LA USA; 6 Department of Structural & Cellular Biology, Tulane University School of Medicine, New Orleans, LA USA; 7 Dental and Oral Medical Center, Kurume University School of Medicine, Fukuoka, Japan; 8 Division of Gross and Clinical Anatomy, Department of Anatomy, Kurume University School of Medicine, Fukuoka, Japan; 9 Department of Neurosurgery and Ochsner Neuroscience Institute, Ochsner Health System, New Orleans, LA USA; 10 Oral and Maxillofacial Surgery, Shizuoka City Shizuoka Hospital, Shizuoka, Japan; 11 Department of Oral and Maxillofacial Surgery, Faculty of Medicine, Dentistry and Pharmaceutical Sciences, Okayama University, Okayama, Japan; 12 Department of Cell Biology, Nagasaki University Graduate School of Biomedical Sciences, Nagasaki, Japan; 13 Department of Anatomical Sciences, St. George’s University, St. George’s, Grenada; 14 Department of Surgery, Tulane University School of Medicine, New Orleans, LA USA; 15 University of Queensland, Brisbane, Australia; 16 Department of Oral and Maxillofacial Radiology, Okayama University Hospital, Okayama, Japan

**Keywords:** Anatomy, Omohyoid muscle, Computed tomography, Neck dissection, Vertebra, Cancer

## Abstract

**Purpose:**

The current supraomohyoid neck dissection (SOHND) is performed above the omohyoid muscle to dissect levels I, II, and III in the levels of cervical lymph nodes. However, the anatomical boundary between levels III and IV is the inferior border of the cricoid cartilage. We investigated the anatomical relationship between the omohyoid muscle and cricoid cartilage using contrast-enhanced CT (CE-CT) images to assess the validity of the current SOHND.

**Methods:**

CE-CT images of the head and neck regions in patients were reviewed. The patients were divided into two groups: “malignant tumors” and “others”. The vertebral levels corresponding to the positions of anatomical structures such as the intersection of the omohyoid muscle and internal jugular vein (OM-IJ), and the inferior border of the cricoid cartilage (CC), were recorded.

**Results:**

The OM-IJ was located around the seventh cervical to the first thoracic vertebra. There was a significant difference between the malignant tumor and others groups in females (*p* = 0.036). The CC was located around the sixth to seventh cervical vertebrae. There was a significant sex difference in each group (malignant tumor: *p* < 0.0001; others: *p* = 0.008). Both sexes tended to have lower OM-IJ than CC, and females had significantly lower OM-IJ than males.

**Conclusion:**

This study provides clear anatomical evidence showing the difference between the SOHND dissection area and levels I, II, and III. It could be considered that in most cases SOHND invades level IV, not just levels I, II, and III, especially in female patients.

## Introduction

A neck dissection is a widely accepted surgical procedure for metastatic cancers of the head and neck region. A prophylactic selective neck dissection (SND) is recommended to treat the patients with clinically node-negative. The SND is considered as effective as radical neck dissection (RND). Many modified techniques have been reported depending on the purpose/diagnosis [2, 3, 5]. A supraomohyoid neck dissection (SOHND), a term first used by Byers in 1985 in his report of a large group (1372 cases of modified neck dissection), is a modified RND technique that removes the tissue on and above the omohyoid muscle en bloc [2]. Indication for the SOHND is oral cancer or oropharynx cancer with no preoperative evidence of neck lymph node metastases [2, 8, 9]. A SOHND is classed as SND of lymph nodes in levels I, II, and III of the neck [4, 7]. When the SOHND was first reported by Byers in 1985, there was little anatomical/clinical evidence to support it [2]. There is now much clinical evidence for its efficacy [18, 20].

According to the levels of cervical lymph nodes with anatomical boundaries, the superior and inferior borders of level III are “horizontal plane defined by inferior body of hyoid” and “horizontal plane defined by the inferior border of the cricoid cartilage,” respectively [15]. There is no description of the omohyoid muscle to define the border of levels III and IV. Interestingly, Shah et al. located level III lymph nodes on the level where the omohyoid muscle crosses the internal jugular chain [16]. Anatomically, the level of the omohyoid muscle and cricoid cartilage should not be related, but the hyoid bone inserts in the superior belly of the omohyoid muscle. Thus, the position of the omohyoid muscle and the border between levels III and IV could be inconsistent, although many surgeons still believe the SOHND is the procedure for removing lymph nodes from levels I, II, and III [4, 7]. This is probably because the jugulo-omohyoid lymph nodes, which lie on or right above the intermediate tendon of the omohyoid muscle, are considered one of the main targets of this procedure [19].

Many clinical reports show that patients preoperatively diagnosed with no metastatic lymph nodes could have pathologically positive nodes. According to Spiro et al., 25% of 248 SOHND clinically negative lymph nodes were pathologically positive [18]. In those cases, a slight difference between the positions of the omohyoid muscle and cricoid cartilage could change the patient’s prognosis. To predict a better outcome, precise management of the cervical lymph nodes is required with accurate anatomical description. The area of the neck dissection could differ greatly between cases with lower omohyoid muscle than cricoid cartilage and those with higher omohyoid muscle than cricoid cartilage.

Our aim in this study was to investigate the anatomical relationship between the omohyoid muscle and cricoid cartilage using contrast-enhanced CT (CE-CT) images to assess the validity of the current SOHND for future clinical studies.

## Materials and methods

### Study subjects

We retrospectively reviewed CE-CT images of the head and neck regions in patients who were referred to our institution from January 2019 to December 2021. CE-CT images taken at the patient’s initial visit or before operation were included. The patients were divided into two groups: “malignant tumors” and “others” (benign tumor, cyst, inflammation). Sex, age, body height, and weight of each patient were recorded.

### Image preparation

CE-CT images were taken with four types of CT (Aquilion ONE: Canon Medical Systems Corporation, Tochigi, Japan; Aquilion Precision: Canon Medical Systems Corporation; Discovery CT750 HD: GE Healthcare, Milwaukee, WI, USA; SOMATOM Definition Flash: Siemens AG, Munich, Germany). There were three slice thicknesses of CT images: 0.8, 1.00 and 1.25 mm. Axial and sagittal images of the healthy side (i.e. not diseased) were used for accurate measurements. Under the instruction of the oral and maxillofacial radiologists, all patients were positioned in the supine position so that the occlusal plane was parallel to the cross section of scanning when CE-CT images were taken.

### Measurements

**Fig. 1:**
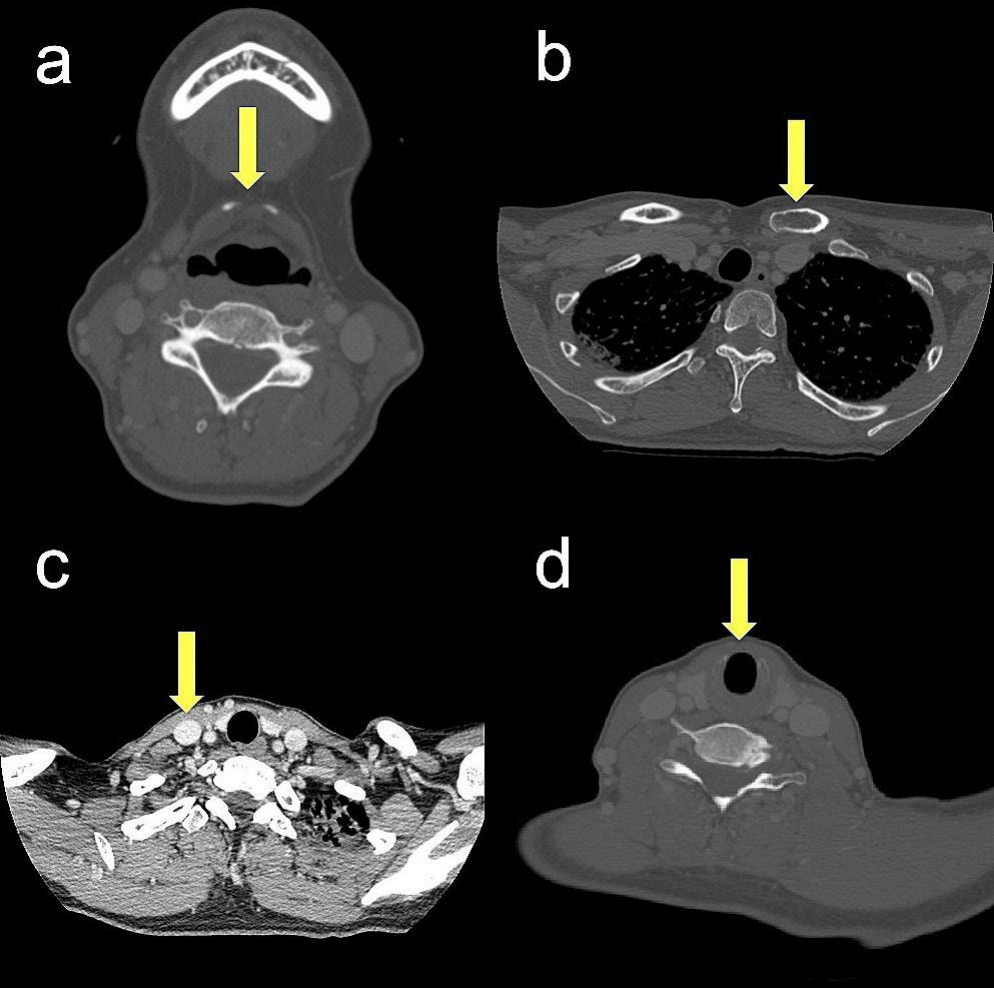
The points of measurement of anatomical structures in the slice of contrast-enhanced CT (CE-CT) images. (**a**) Central inferior border of hyoid bone (HB). (**b**) Superior border of sternal end of clavicle (CL). (**c**) Intersection of omohyoid muscle and internal jugular vein (OM-IJ). (**d**) Inferior border of cricoid cartilage (CC). The OM-IJ was identified using CE-CT images with soft tissue windows, and the other three anatomical structures (HB, CL, and CC) were identified with the bone windows

**Fig. 2:**
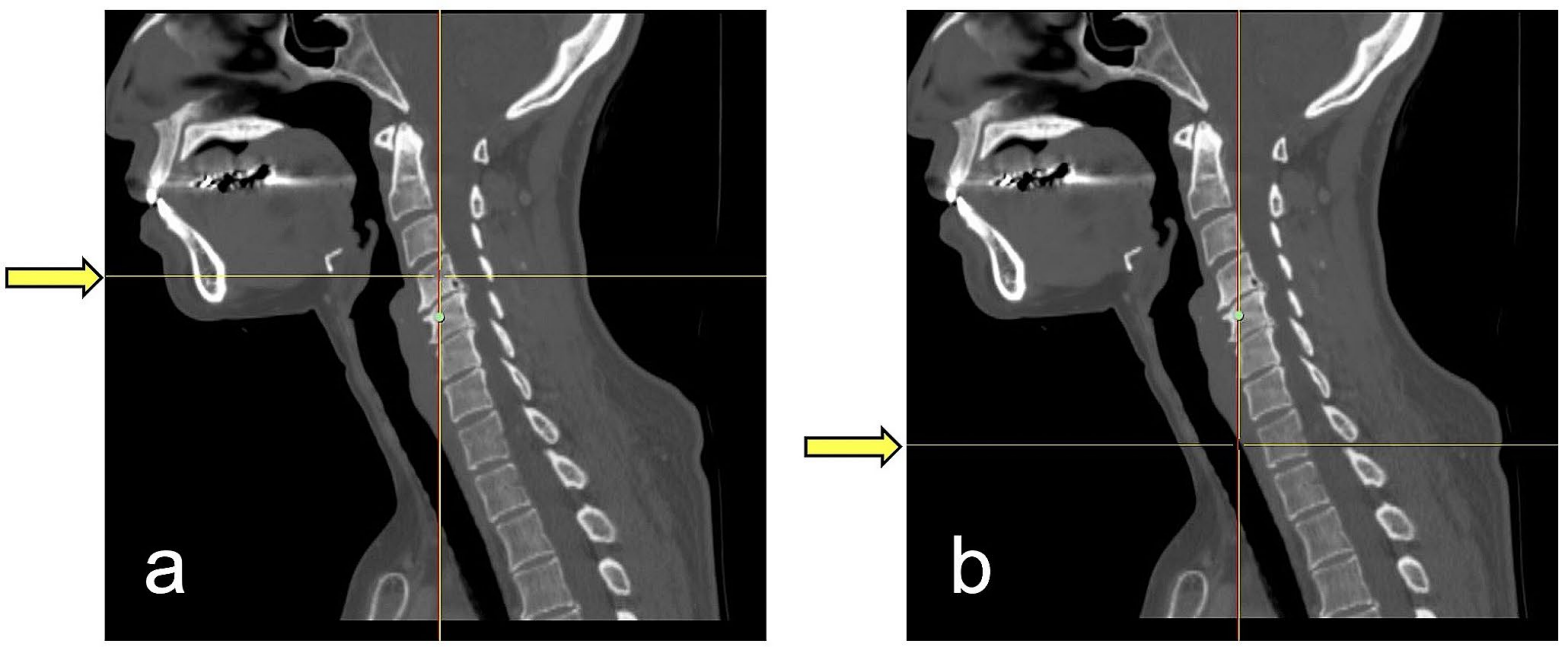
Examples of measurement of the vertebral level corresponding to the slice of CE-CT images of anatomical structures. (**a**) Vertebral level of central inferior border of hyoid bone (HB). The location of HB corresponded to the fourth cervical vertebra in this case. (**b**) Vertebral level of inferior border of cricoid cartilage (CC). The location of CC corresponded to the first thoracic vertebra in this case

The slices of the CE-CT images corresponding to the following positions of anatomical structures were recorded: central inferior border of hyoid bone (HB); superior border of sternal end of clavicle (CL); intersection of omohyoid muscle and internal jugular vein (OM-IJ); inferior border of cricoid cartilage (CC). The OM-IJ was identified using CE-CT images with soft tissue windows, and the other three anatomical structures were identified with bone windows (Fig. 1). In addition, the vertebral levels corresponding to the slice of CE-CT images of these four anatomical structures were noted. The mean vertebral level was calculated when the slice straddled two vertebral bones, and the vertebral level was numbered serially from the first cervical vertebra to simplify analyses (Fig. 2).

**Fig. 3:**
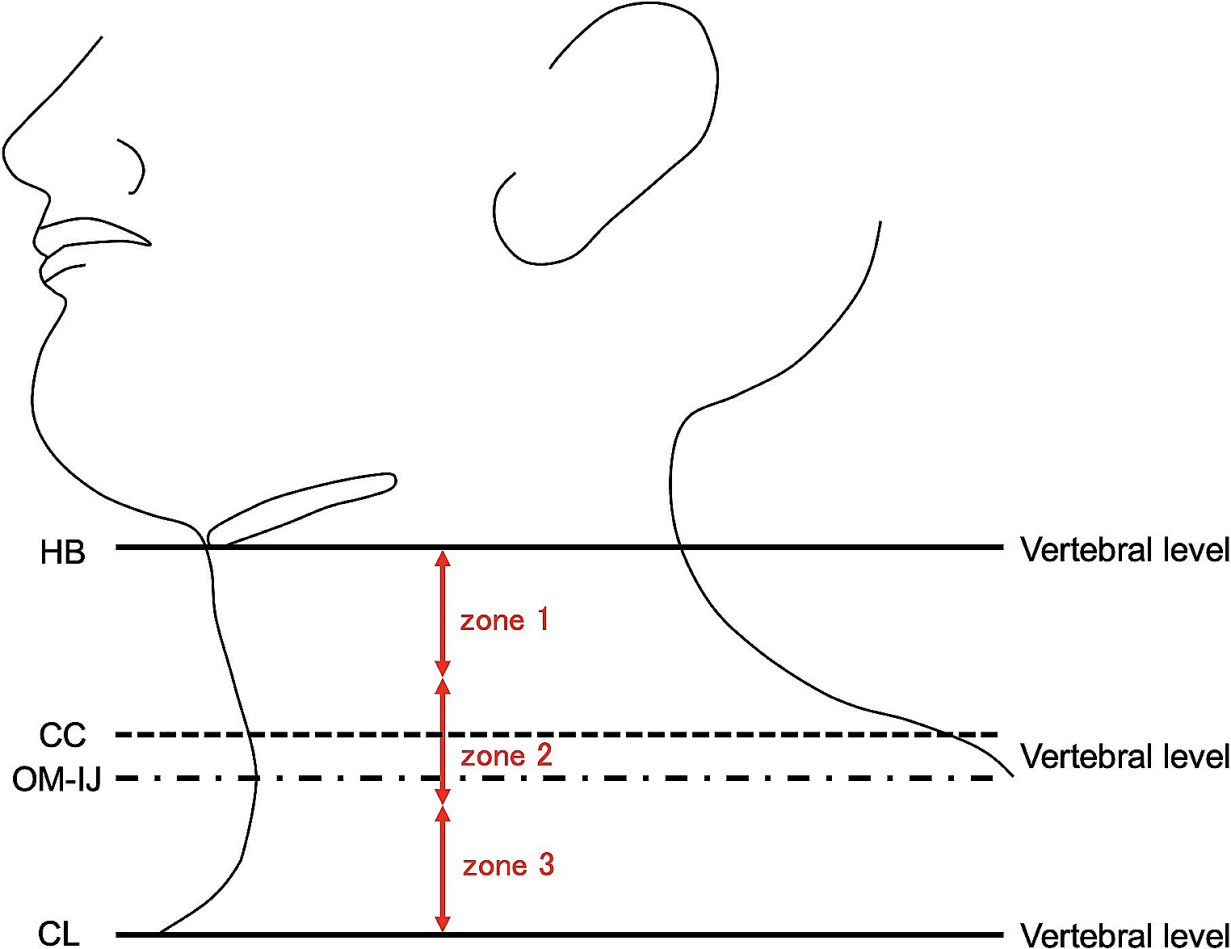
The area from the central inferior border of the hyoid bone (HB) to the superior border of the sternal end of the clavicle (CL) was divided equally into three zones from superior to inferior to locate the intersection of the omohyoid muscle and internal jugular vein (OM-IJ) or inferior border of cricoid cartilage (CC)

The area from the HB to the CL was divided equally into three zones from superior to inferior, i.e., zone 1, zone 2, and zone 3, to identify the locations of the OM-IJ and CC. The positional relationship between the OM-IJ and CC in relation the level of the lymph node regions of the neck, and the distance between them, were also recorded (Fig. 3).

One oral and maxillofacial radiologist performed all the measurements of the CE-CT images. Measurements were taken twice over a one-month interval.

### Statistical analysis

Intraobserver agreement between the first and second measurements was measured using kappa values and classed into five grades: poor agreement (< 0.20); fair agreement (0.21–0.40); moderate agreement (0.41–0.60); good agreement (0.61–0.80); excellent agreement (0.81-1.00). The significance of differences among measurements was measured by the Mann-Whitney U test and the Pearson’s chi-square test using SPSS (version 27, IBM, NY, USA). *p* < 0.05 was considered statistically significant.

The present study protocol was approved by Okayama University Ethics Committee (approval No. 2203-007), and the study was performed in accordance with the requirements of the Declaration of Helsinki (64th WMA General Assembly, Fortaleza, Brazil, October 2013). Informed consent was obtained from all patients for inclusion in the study.

## Results

One hundred and four patients were evaluated. Fifty-five (25 males and 30 females) had “malignant tumors” and 49 (22 males and 27 females) were “others” (Table 1). The mean age was 64.5 years (range 19–93); the mean age of “malignant tumors” was 69.0 years (range 30–93) and of “others” was 57.5 years (range 19–89). The mean height and weight were 159.0 cm (range 131.6–181.0 cm) and 54.9 kg (range 29.4–93.4 kg), respectively. In the “malignant tumor” group, the corresponding values were 157.3 cm (range 135.5–180.0 cm) and 51.1 kg (range 29.4–82.5 kg); in the “others” group they were 160.8 cm (range 131.6–181.0 cm) and 59.2 kg (range 34.1–93.4 kg), respectively.

**Table 1:** Distribution of numbers of patients

	No. of patients	No. of malignant tumors	No. of others
Male	47	25	22
Female	57	30	27
Total	104	55	49

### Kappa values

The kappa values for the slice of CE-CT images of the HB, CL, OM-IJ, and CC were 0.981, 0.972, 0.969, and 0.976, respectively. Those for the vertebral level of the HB, CL, OM-IJ, and CC were 0.873, 0.741, 0.687, and 0.756, respectively. Those for the zone of the OM-IJ and CC were 0.670 and 0.800, respectively. Those for the positional relationship between the OM-IJ and CC in relation to the level of the lymph node regions of the neck, and the distance, were 0.928 and 0.865, respectively. All kappa values showed good to excellent intraobserver agreement. The second measurements were used for subsequent analysis.

### The vertebral level of the HB

**Fig. 4:**
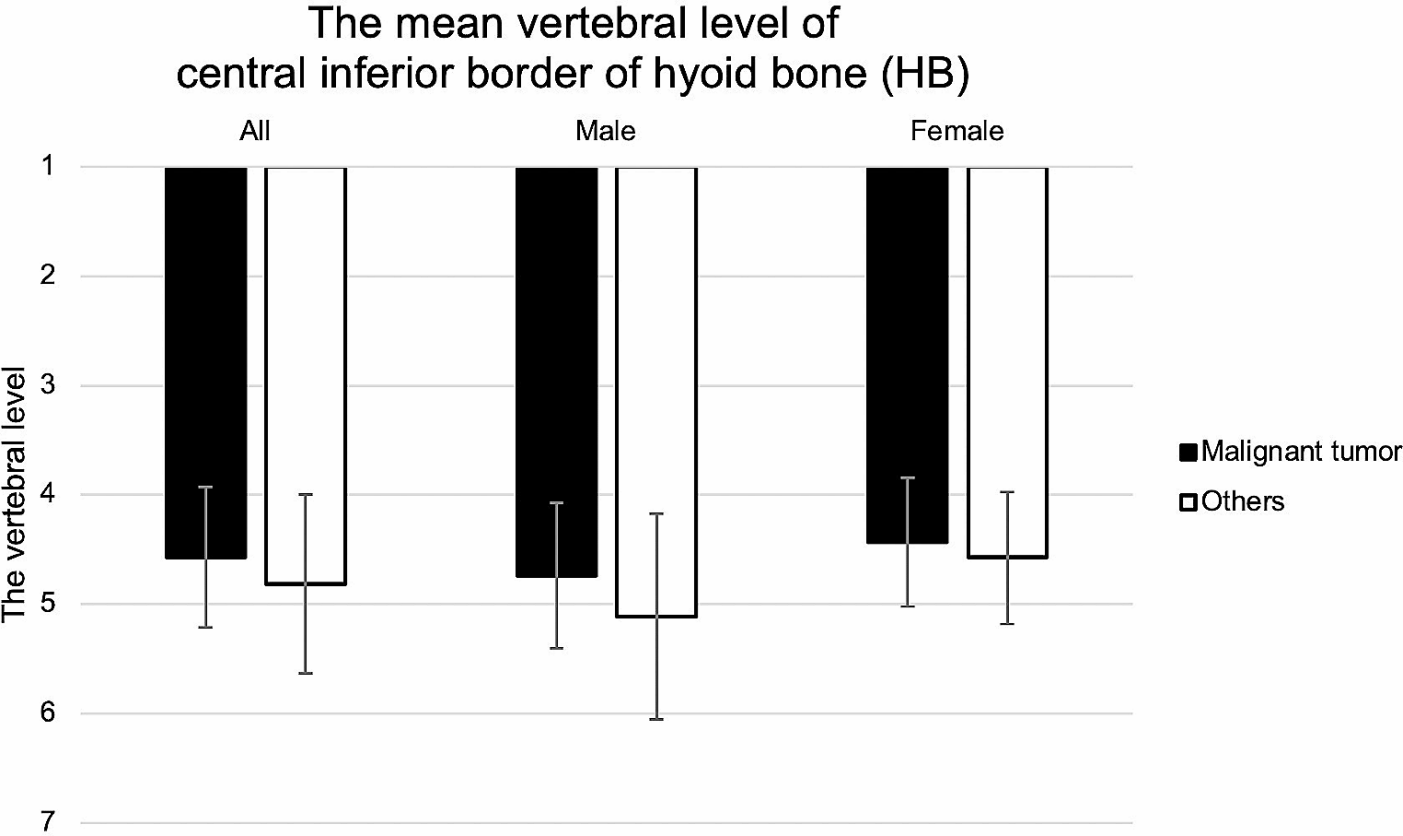
Mean vertebral level of central inferior border of hyoid bone (HB). The HB was located around the fourth to fifth cervical vertebra. Males tended to have slightly lower HB than females. There were no significant group of sex differences (*p* > 0.05)

The mean vertebral levels of the HB in the malignant tumors and others groups were 4.57 and 4.82, respectively (males: 4.74 and 5.11; females: 4.43 and 4.57). Hence, the HB was located around the fourth to fifth cervical vertebra. Males tended to have slightly lower HB than females. There were no significant sex or group differences (*p* > 0.05, Mann-Whitney U test) (Fig. 4).

### The vertebral level of the CL

**Fig. 5:**
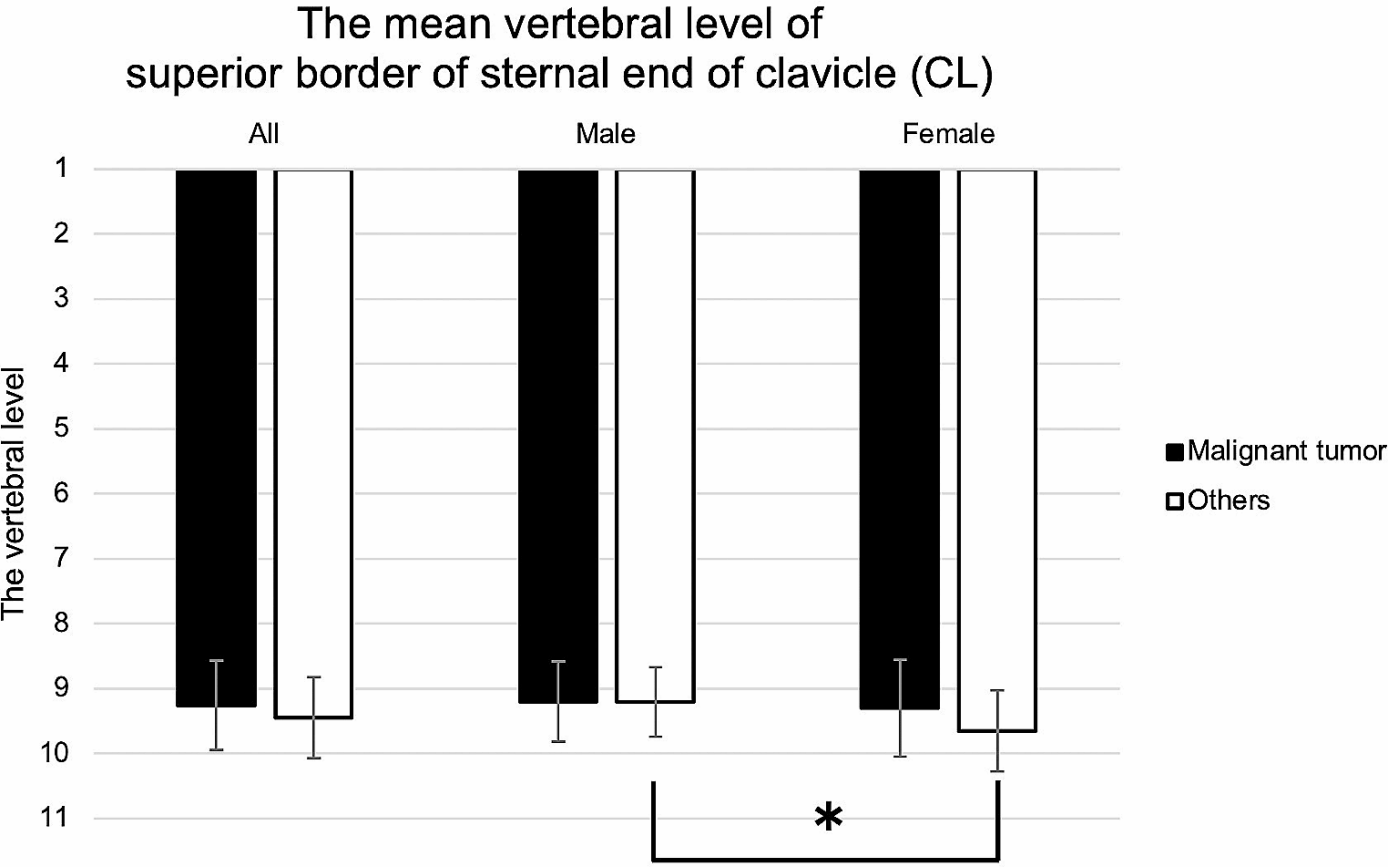
Mean vertebral level of superior border of sternal end of clavicle (CL). The CL was located around the second to third thoracic vertebra. Females tended to have slightly lower CL than males. There was significant difference between the sexes in others (*p = 0.02)

The mean vertebral levels of the CL in the malignant tumors and others groups were 9.25 and 9.46, respectively (males: 9.20 and 9.20; females: 9.30 and 9.65). Hence, the CL was located around the second to third thoracic vertebrae. Females tended to have slightly lower CL than males. There was significant sex difference in the others group (*p* = 0.02, Mann-Whitney U test) (Fig. 5).

### The vertebral level and zone of the OM-IJ

**Fig. 6:**
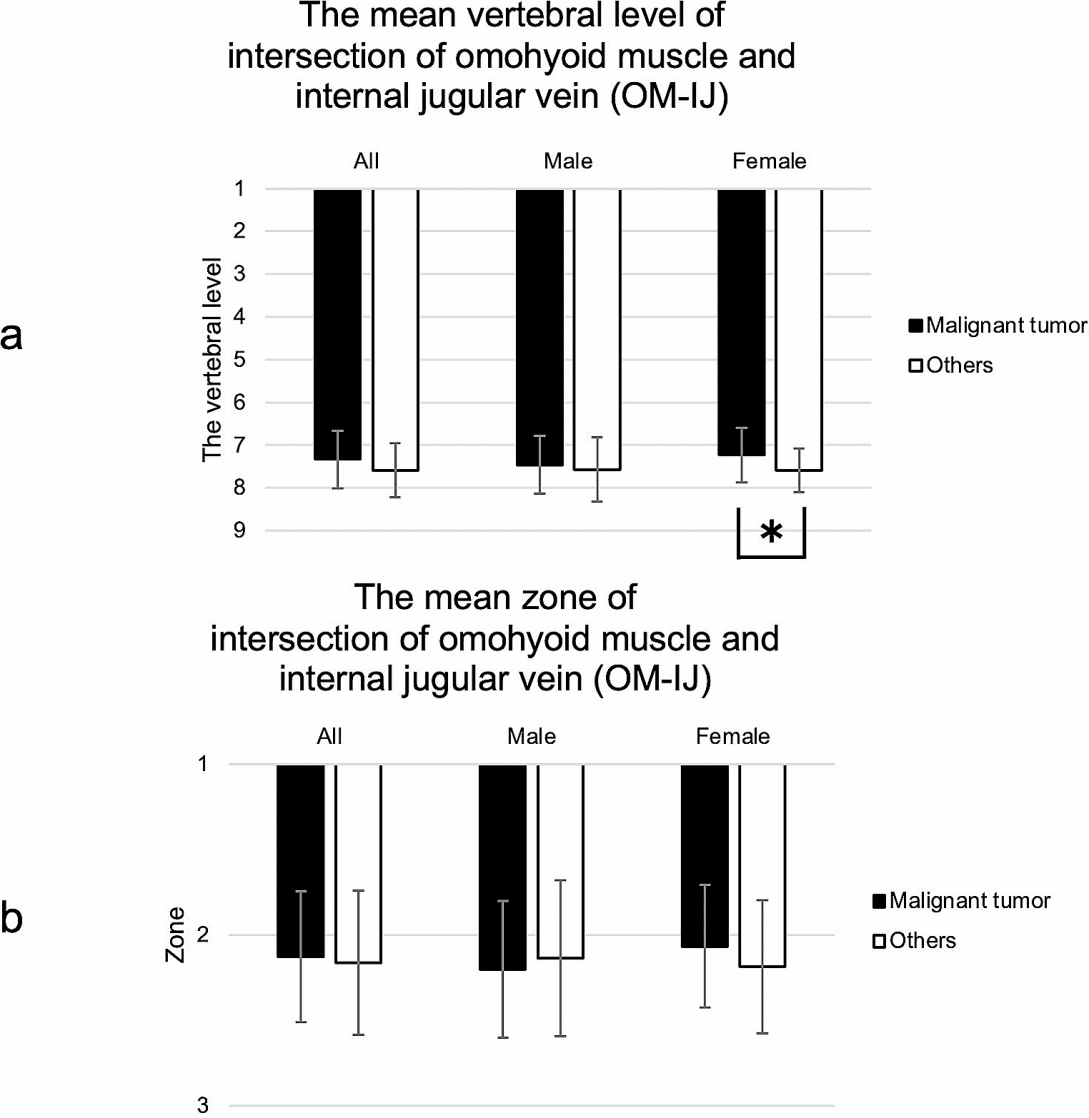
(**a**) The mean vertebral level and zone of intersection of omohyoid muscle and internal jugular vein (OM-IJ). The OM-IJ was located around the seventh cervical vertebra to the first thoracic vertebra. There was significant difference between malignant tumors and others in females (*p = 0.036). (**b**) OM-IJ was also located in zone 2. There were no significant group of sex differences (*p* > 0.05)

The mean vertebral level of the OM-IJ in the malignant tumors and others groups were 7.34 and 7.58, respectively (males: 7.46 and 7.57; females: 7.23 and 7.59). Hence, the OM-IJ was located around the seventh cervical to the first thoracic vertebra. There was a significant group difference in females (*p* = 0.036, Mann-Whitney U test). The mean zones of the OM-IJ in the malignant tumors and others groups were 2.13 and 2.16, respectively (males: 2.20 and 2.14; females: 2.07 and 2.19). Hence, most OM-IJs were located in zone 2. There were no significant sex or group differences (*p* > 0.05, Mann-Whitney U test) (Fig. 6).

### The vertebral level and zone of the CC

The mean vertebral levels of the CC in the malignant tumors and others groups were 6.79 and 6.70, respectively (males: 7.34 and 7.07; females: 6.33 and 6.41). Hence, the CC was located around the sixth to seventh cervical vertebrae. Males tended to have lower CC than females. There was a significant sex difference in each group (malignant tumor: *p* < 0.0001; others: *p* = 0.008, Mann-Whitney U test).

**Fig. 7:**
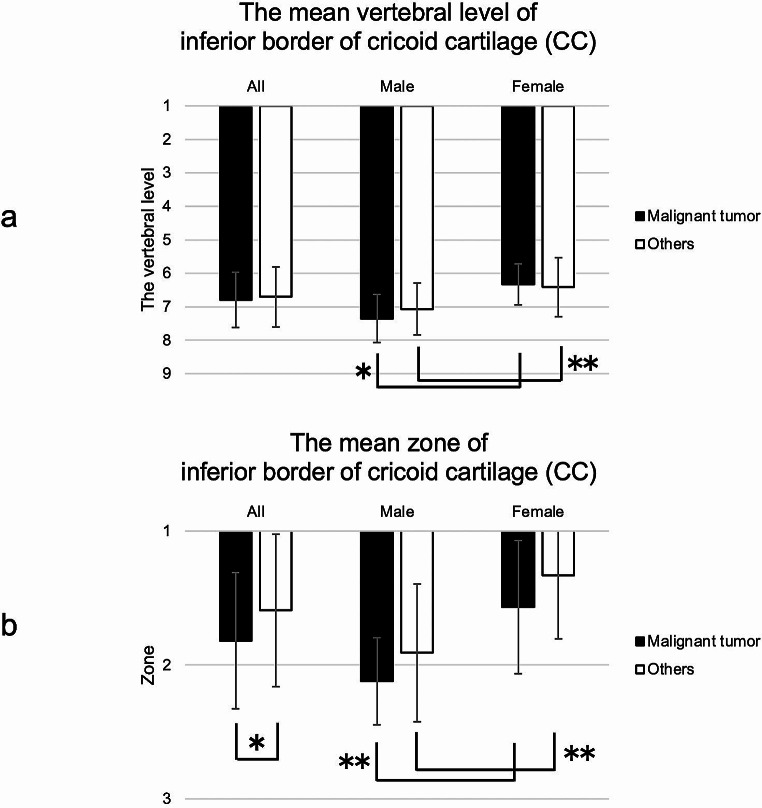
(**a**) The mean vertebral level and zone of inferior border of cricoid cartilage (CC). The CC was located around the sixth cervical vertebra to the first thoracic vertebra. Males tended to have lower CC than females. There was significant sex difference in each group (malignant tumor: *p < 0.0001; others: **p = 0.008). (**b**) Most CC were located in zones 1–2. There was significant difference between malignant tumors and others in all patients (*p = 0.03). There was significant sex difference in each group (malignant tumor: **p < 0.0001; others: **p < 0.0001)

The mean zones of the CC in the malignant tumor and others groups were 1.82 and 1.59, respectively (males: 2.12 and 1.91; females: 1.57 and 1.33). Hence, most CCs were located in zones 1–2. There was significant group difference in all patients (*p* = 0.03, Mann-Whitney U test). There was a significant sex difference in each group (malignant tumors: *p* < 0.0001; others: *p* < 0.0001, Mann-Whitney U test) (Fig. 7).

### Positional relationship between the OM-IJ and CC

**Fig. 8:**
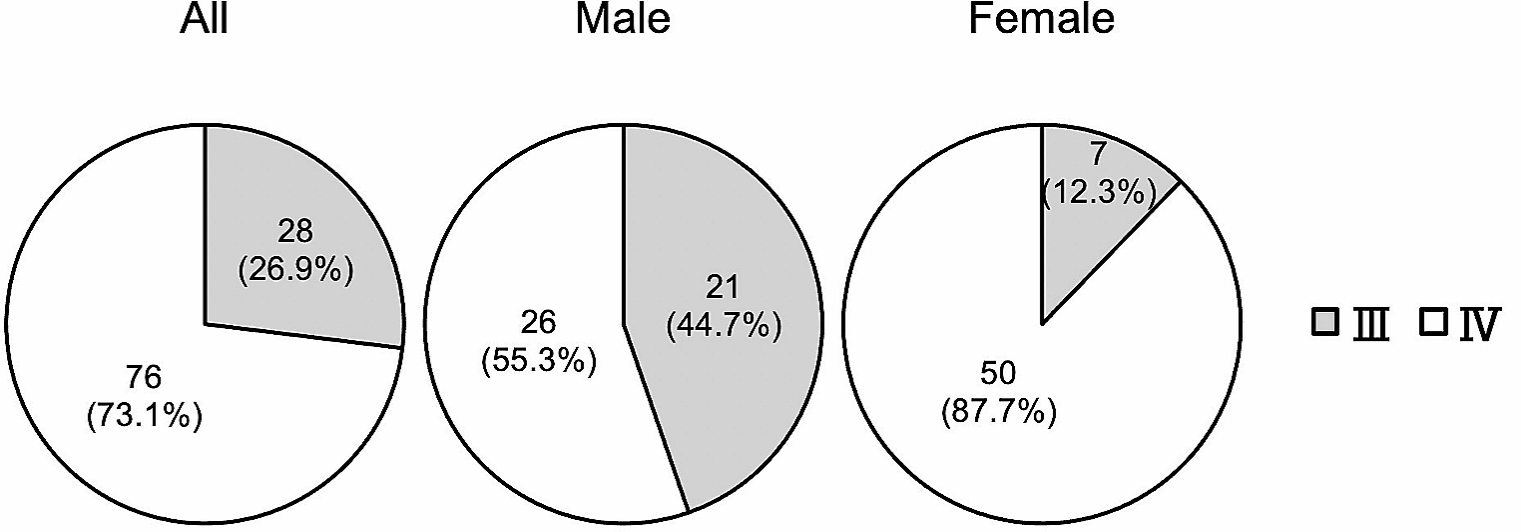
Distribution of the positional relationship between intersection of omohyoid muscle and internal jugular vein (OM-IJ) and inferior border of cricoid cartilage (CC) in relation to the level of the lymph node regions of the neck. Males with OM-IJ in level III and females with OM-IJ in level IV were significantly higher, and males with OM-IJ in level IV and females with OM-IJ in level III were significantly lower (*p*< 0.0001)

Among the 104 patients, 28 (26.9%) had higher OM-IJ and 76 (73.1%) had lower OM-IJ than CC. In other words, 28 patients had the OM-IJ in level III and 76 in level IV. Of the 47 males, 21(44.7%) had the OM-IJ in level III and 26 (55.3%) in level IV. Of the 57 females, seven (12.3%) had the OM-IJ in level III and 50 (87.7%) in level IV. Males with the OM-IJ in level III and females with it in level IV were significantly higher, and males with the OM-IJ in level IV and females with it in level III were significantly lower (p < 0.0001, Pearson’s chi-square test) (Fig. 8).

**Fig. 9:**
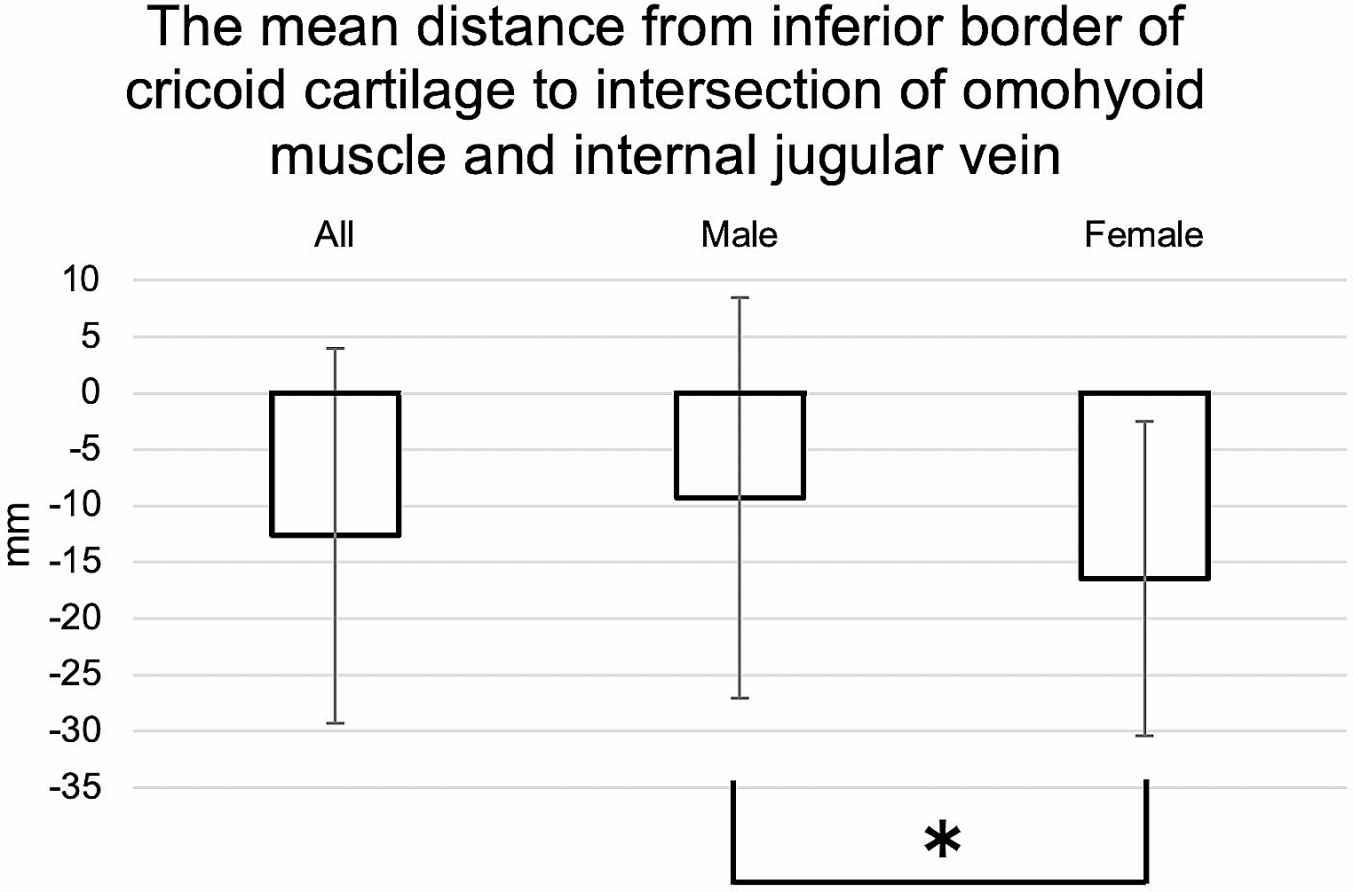
The mean distance from inferior border of cricoid cartilage (CC) to intersection of omohyoid muscle and internal jugular vein (OM-IJ). Both sexes tended to have OM-IJ lower than CC, and females had significantly lower OM-IJ than males (*p < 0.0001)

The distance from the CC to the OM-IJ ranged from − 63.75 to 22.0 mm (mean − 12.66 mm; males: -9.29 mm, females: -16.45 mm). Both sexes tended to have lower OM-IJ than CC, and females had significantly lower OM-IJ than males (*p* < 0.0001, Mann-Whitney U test) (Fig. 9).

## Discussion

The deep cervical lymph nodes surrounding the internal jugular vein (IJV) constitute a major conduit for metastases of head and neck cancers. The positions of landmark structures could change the range of neck dissection. To standardize the surgical procedure, reliable anatomical landmarks are necessary. In order to compare the positions of the anatomical structures, we used the vertebral level of each structure, i.e., HB, CL, OM-IJ, and CC, which is necessary for discussing levels III and IV.

### Vertebral levels of landmark structures

The vertebral level of the HB was C4-5 in this study, in agreement with the CT studies by Badshah et al. (C4) and Mirjalili et al. (C4) [1, 12]. The vertebral level of the CL ranged from Th2 to Th3. This was consistent with the MRI study by Lakshmanan et al., which showed 62.2% of sternal notches positioned between Th2 and Th3. The OM-IJ level in the present study was C7 to Th1; this has not been investigated in any previous anatomical or radiological study [10]. The level of the OM-IJ could be very close to the clavicle in some patients. The CC was positioned at C6-C7 level, in agreement with Mirjalili et al. [12].

### Positional relationship of the OM-IJ and CC

The level of the OM-IJ was in zone 2 (also vertebral level C7-Th1) and that of the CC was in zone 1–2 (also vertebral level C6-7). The OM- IJ was located inferior to the CC. When the CC was considered the border between levels III and IV, the OM-IJ was in level IV in 73% of cases (88% of females and 55% of males). These results show that in most cases SOHND invades level IV, not just levels I, II, and III. Especially in female patients, the OM-IJ was more frequently located at level IV. As the dissected area could be too invasive, the surgeons should recognize the positional difference between OM- IJ and CC.

### Anatomical consideration of the omohyoid muscle and jugulo-omohyoid lymph nodes

The omohyoid muscle originates from the superior border of the scapula to the hyoid bone via the intermediate tendon. It is also connected to the clavicle by a fascial sling [13], which is believed to be part of the deep cervical fascia, i.e., fusion of the investing and pretracheal layers [11, 14]. This sling could affect the position of the omohyoid muscle by the motion of the clavicle. Frequent anatomical variations of the omohyoid muscle have been reported, e.g., double omohyoid, clavicular attachment [6, 14, 17]. An anomaly can change the dissection area of SOHND. The jugulo-omohyoid group of lymph nodes lies on or above the intermediate tendon of the omohyoid muscle [19]. However, anatomical evidence about this nodal group is scant.

### Future direction

A SOHND is generally considered the prophylactic neck dissection of levels I, II, and III for oral cancers with no preoperative evidence of neck lymph node metastases [7]. The difference between the OM-IJ and CC should be considered. On the basis of current evidence, using the cricoid cartilage as the border of levels III and IV seems less reliable than using the omohyoid muscle. Practically, it could be better to use the omohyoid muscle as an anatomical landmark. Therefore, mapping the lymph nodes around the OM-IJ is extremely important.

Taking account of cases in which cancer-positive lymph nodes were removed by SOHND, standardizing the dissection level is a task to be addressed urgently.

## Conclusions

This study provides anatomical evidence clearly showing the difference between the SOHND dissection area and levels I, II, and III, especially in female patients. We believe the level classification is still necessary for surgeons, but evidence-based anatomical structures need to be used as the border between levels III and IV. In future studies, mapping the lymph nodes associated with the cricoid cartilage, omohyoid muscle, and IJV could be useful for determining the border between levels III and IV.

### Limitation

All patients in this study were Japanese, so racial differences could have affected the results. Although we defined the intersection of the omohyoid muscle and internal jugular vein as the inferior border of level III, this could differ among institutions. We used three kinds of slice thickness of CT image. The position of the patients for the CT scan might not have been completely the same. Therefore, there could have been small errors, especially in the actual measurements.

## Data Availability

No datasets were generated or analysed during the current study.
