# Correction: Radiological assessment of the dissection area in supraomohyoid neck dissection

**DOI:** 10.1007/s00276-024-03493-9

**Published:** 2024-10-10

**Authors:** Yohei Takeshita, Joe Iwanaga, Yoshio Ohyama, Soichiro Ibaragi, Yuki Matsushita, R. Shane Tubbs, Norio Kitagawa, Toshiyuki Kawazu, Miki Hisatomi, Shunsuke Okada, Mamiko Fujikura, Junichi Asaumi

**Affiliations:** 1 Department of Oral and Maxillofacial Radiology, Faculty of Medicine, Dentistry and Pharmaceutical Sciences, Okayama University, 2-5-1 Shikata-cho, Kita-ku, Okayama, 700-8558 Japan; 2 Clinical Anatomy Research Association in Oral and Maxillofacial Surgery, Fukuoka, Japan; 3 Department of Oral and Maxillofacial Anatomy, Graduate School of Medical and Dental Sciences, Tokyo Medical and Dental University, Tokyo, Japan; 4 Department of Neurosurgery, Tulane Center for Clinical Neurosciences, Tulane University School of Medicine, New Orleans, LA USA; 5 Department of Neurology, Tulane Center for Clinical Neurosciences, Tulane University School of Medicine, New Orleans, LA USA; 6 Department of Structural & Cellular Biology, Tulane University School of Medicine, New Orleans, LA USA; 7 Dental and Oral Medical Center, Kurume University School of Medicine, Fukuoka, Japan; 8 Division of Gross and Clinical Anatomy, Department of Anatomy, Kurume University School of Medicine, Fukuoka, Japan; 9 Department of Neurosurgery and Ochsner Neuroscience Institute, Ochsner Health System, New Orleans, LA USA; 10 Oral and Maxillofacial Surgery, Shizuoka City Shizuoka Hospital, Shizuoka, Japan; 11 Department of Oral and Maxillofacial Surgery, Faculty of Medicine, Dentistry and Pharmaceutical Sciences, Okayama University, Okayama, Japan; 12 Department of Cell Biology, Nagasaki University Graduate School of Biomedical Sciences, Nagasaki, Japan; 13 Department of Anatomical Sciences, St. George’s University, St. George’s, Grenada; 14 Department of Surgery, Tulane University School of Medicine, New Orleans, LA USA; 15 University of Queensland, Brisbane, Australia; 16 Department of Oral and Maxillofacial Radiology, Okayama University Hospital, Okayama, Japan

**Correction: Surgical and Radiologic Anatomy (2024) 46:1643–1652** 10.1007/s00276-024-03453-3

In this article first paragraph has been updated under the Positional relationship section and the Fig. 8 legend were wrongly appeared.

The correct paragraph as given below,

Positional relationship between the OM-IJ and CC

Among the 104 patients, 28 (26.9%) had higher OM-IJ and 76 (73.1%) had lower OM-IJ than CC. In other words, 28 patients had the OM-IJ in level III and 76 in level IV. Of the 47 males, 21(44.7%) had the OM-IJ in level III and 26 (55.3%) in level IV. Of the 57 females, seven (12.3%) had the OM-IJ in level III and 50 (87.7%) in level IV. Males with the OM-IJ in level III and females with it in level IV were significantly higher, and males with the OM-IJ in level IV and females with it in level III were significantly lower (p < 0.0001, Pearson’s chi-square test) (Fig. 8).

The Correct Fig. 8 legend as given below,

**Fig. 8:**
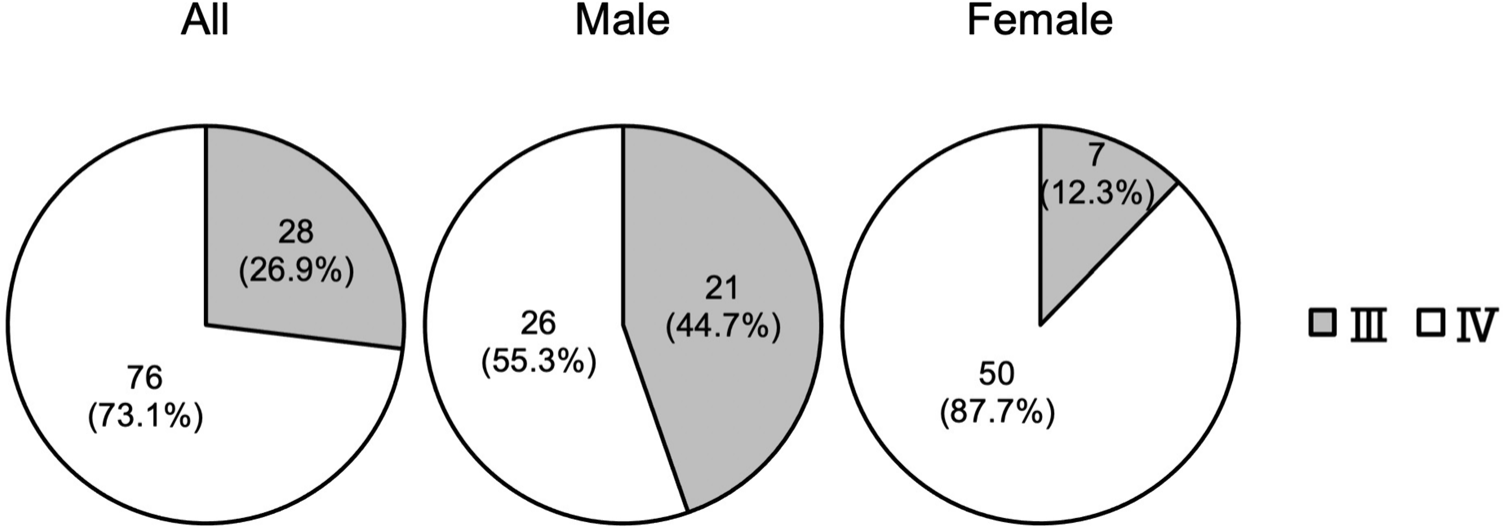
Distribution of the positional relationship between intersection of omohyoid muscle and internal jugular vein (OM-IJ) and inferior border of cricoid cartilage (CC) in relation to the level of the lymph node regions of the neck. Males with OM-IJ in level III and females with OM-IJ in level IV were significantly higher, and males with OM-IJ in level IV and females with OM-IJ in level III were significantly lower (*p* < 0.0001)

